# Genome-Wide Association Study Identifies NBS-LRR-Encoding Genes Related with Anthracnose and Common Bacterial Blight in the Common Bean

**DOI:** 10.3389/fpls.2017.01398

**Published:** 2017-08-09

**Authors:** Jing Wu, Jifeng Zhu, Lanfen Wang, Shumin Wang

**Affiliations:** Institute of Crop Sciences, Chinese Academy of Agricultural Sciences Beijing, China

**Keywords:** common bean, NBS-LRR, disease resistance genes, anthracnose, common bacterial blight, association study

## Abstract

Nucleotide-binding site and leucine-rich repeat (NBS-LRR) genes represent the largest and most important disease resistance genes in plants. The genome sequence of the common bean (*Phaseolus vulgaris* L.) provides valuable data for determining the genomic organization of NBS-LRR genes. However, data on the NBS-LRR genes in the common bean are limited. In total, 178 NBS-LRR-type genes and 145 partial genes (with or without a NBS) located on 11 common bean chromosomes were identified from genome sequences database. Furthermore, 30 NBS-LRR genes were classified into Toll/interleukin-1 receptor (TIR)-NBS-LRR (TNL) types, and 148 NBS-LRR genes were classified into coiled-coil (CC)-NBS-LRR (CNL) types. Moreover, the phylogenetic tree supported the division of these PvNBS genes into two obvious groups, TNL types and CNL types. We also built expression profiles of NBS genes in response to anthracnose and common bacterial blight using qRT-PCR. Finally, we detected nine disease resistance loci for anthracnose (ANT) and seven for common bacterial blight (CBB) using the developed NBS-SSR markers. Among these loci, NSSR24, NSSR73, and NSSR265 may be located at new regions for ANT resistance, while NSSR65 and NSSR260 may be located at new regions for CBB resistance. Furthermore, we validated NSSR24, NSSR65, NSSR73, NSSR260, and NSSR265 using a new natural population. Our results provide useful information regarding the function of the NBS-LRR proteins and will accelerate the functional genomics and evolutionary studies of NBS-LRR genes in food legumes. NBS-SSR markers represent a wide-reaching resource for molecular breeding in the common bean and other food legumes. Collectively, our results should be of broad interest to bean scientists and breeders.

## Introduction

The common bean is an important global pulse crop that can be used for human consumption or animal fodder. However, one of the main problems that limits bean production in every country is disease, such as anthracnose (Melotto et al., 2000; Singh and Schwartz, 2010), angular leaf spot (Schwartz et al., 1981; de Jesus et al., 2001; Bassanezi et al., 2002), powdery mildew (Trabanco et al., 2012), or common bacterial blight (Opio et al., 1996; Fourie, 2002; Zamani et al., 2011). Cultivating resistant varieties is the most economical and effective approach for controlling common bean diseases and is an important goal for common bean breeders. In recent years, the loss of disease-resistant varieties has proven to be a challenge for common bean production. Therefore, the discovery of new disease resistance genes represents a vital task and an important precondition for breeding disease-resistant varieties of the common bean.

Nucleotide-binding site (NBS)-leucine-rich repeat (LRR) genes are the most important type of disease resistance genes in plants. The NBS domain participates in the transduction of the disease resistance signal in combination with ATP or GTP, and it contains Kinase 1, Kinase 2, and Kinase 3 and a conserved HD motif, which can be used to identify pathogenic effects on host cells (Meyers et al., 2003; Tameling et al., 2006). NBS-LRR are included two major classes: the first class contains a Toll/interleukin-1 receptor (TIR) motif at the C-terminus, and the second class contains the coiled-coil (CC) motif at the C-terminus (Meyers et al., 2003; McHale et al., 2006) In addition, the highly diverse LRR domain was identified at the N-terminal region (Bent, 1996; Martin et al., 2003). Many studies have shown that NBS-encoding proteins play a necessary role in plant disease resistance to diverse pathogens (Sekhwal et al., 2015). To date, more than 50 NBS genes have been cloned, and exhibited disease resistance functions (Sekhwal et al., 2015). Examples include the stripe rust resistance gene *Yr10* (CC-NBS-LRR) in wheat (Liu et al., 2014), the stem rust resistance gene *Sr33* (CC-NBS-LRR) in wheat (Periyannan et al., 2013), the powdery mildew resistance gene *Mla1* (CC-NBS-LRR) in barley (Zhou et al., 2001), the flax rust resistance gene *L6* (TIR-NBS-LRR) and *M* (TIR-NBS-LRR) genes in flax (Lawrence et al., 1995; Anderson et al., 1997), the fungal pathogen *Peronospora parasitica* resistance gene *Rpp5* (TIR-NBS-LRR) in *Arabidopsis* (Noel et al., 1999), the tobacco mosaic virus resistance gene *N* (TIR-NBS-LRR) in tobacco (Whitham et al., 1994), the bacterial blight-resistance genes *Xa1* and *xa5* (NBS-LRR) in rice (Yoshimura et al., 1998; Iyer and McCouch, 2004), the bacterial and downy mildew resistance gene *RPS5* (NBS-LRR) in *Arabidopsis* (Warren et al., 1998), and the bacterial wilt resistance gene *RRS1* (WRKY-TIR-NBS-LRR) in *Arabidopsis* (Deslandes et al., 2002). However, research on the NBS gene family in the common bean is weak. A previous report revealed that the Co-2 locus contains sequences encoding both NBS-LRR genes (Creusot et al., 1999), JA1tr has been reported to belong to the CC-NBS-LRR class of plant disease R genes (Ferrier-Cana et al., 2005), and the viral resistance gene *RT4-4* is a member of the TIR-NBS-LRR family (Seo et al., 2006). In addition, the bean common mosaic virus resistance *I* gene is located on Pv02 containing a cluster of NBS-LRR-type genes (Vallejos et al., 2006). Many TIR and non-TIR sequences in the common bean were obtained from conserved sequences in the NBS domains of *Medicago truncatula* (Garzon et al., 2013). In conclusion, information on NBS genes in the common bean is limited. Therefore, we should accelerate the identification of the NBS family, which will be beneficial to research into the resistance genes in the common bean.

Anthracnose (ANT) and common bacterial blight (CBB) are important diseases that limit bean production (Singh and Schwartz, 2010). More than 22 resistance genes for ANT (Ferreira et al., 2013; Campa et al., 2014; Gonzalez et al., 2015; Perseguini et al., 2016; Zuiderveen et al., 2016) and 26 resistance quantitative trait loci (QTLs) for CBB (Miklas et al., 2006; Liu et al., 2008; Shi et al., 2011; Xie et al., 2017) have been mapped on all common bean chromosomes. Moreover, genome-wide association studies (GWAS) are powerful and efficient tools for the discovery of novel genes of complex agronomic traits, such as disease resistance against stripe rust, leaf rust, and leaf spot in wheat (Gurung et al., 2014; Zegeye et al., 2014; Iquira et al., 2015; Gao et al., 2016) and the plant hypersensitive defense response genes in maize (Olukolu et al., 2014). Especially in the common bean, Perseguini et al. (2016) and Zuiderveen et al. (2016) identified SNPs or SSRs associated with ANT resistance on 10 common bean chromosomes using genome-wide association studies. Shi et al. (2011) selected SNPs for an association study to map CBB resistance QTL on 11 common bean chromosomes.

In the past 15 years, more than 60 monocot and dicot genomes have been sequenced and assembled (Phytozome v 11.0) in plants, such as in pulses, pigeon pea (Varshney et al., 2012), chickpea (Varshney et al., 2013), common bean (Schmutz et al., 2014; Vlasova et al., 2016), mung bean (Kang et al., 2014), and adzuki bean (Yang et al., 2015). Recently, NBS family sequences within the whole genomes of many plant species have been annotated. In total, 580 complete NBS genes in wheat (Bouktila et al., 2015), 332 NBS-LRR genes in rice (Zhang et al., 2015), 346 NBS-LRR genes in sorghum (Mace et al., 2014), 319 NBS-LRR genes in soybean (Kang et al., 2012), 393 NBS-LRR genes in *A*. *duranensis* (Song et al., 2017), and 437 NBS-LRR genes in *A*. *ipaënsis* (Song et al., 2017) have been detected. However, few studies have investigated the NBS-LRR genes in the common bean at the whole genome level.

Based on annotated genome sequences of the common bean, Andean (G19833) and Mesoamerican (BAT93) genotypes of the common bean genome sequence were found to encompass a 472.5 Mb sequence with 27,197 predicted proteins and a 549.6 Mb sequence with 30,491 protein-coding genes, respectively (Schmutz et al., 2014; Vlasova et al., 2016). These sequences provide an opportunity to explore the characteristics of the NBS genes in the common bean. Thus, the goals of this article are to: (1) identify NBS genes from the common bean; (2) characterize and analyse the genome organization and expression profiles of NBS genes in the common bean; (3) develop SSR markers for these NBS genes; and (4) detect ANT- and CBB-related loci using NBS-SRR markers by the association analysis method.

## Materials and methods

### Plant material, pathogens, and phenotypic evaluation

Two natural populations (183 cultivars and 100 cultivars) were used in the association study (Tables S1, S2). Four common bean genotypes, including Hongyundou (resistant to ANT), Jingdou (susceptible to ANT), HR45 (resistant to CBB), and Bilucaidou (susceptible to CBB), were used to detect the expression profiles of the NBS genes. These seeds can be obtained from the National Gene Bank (China, Beijing). The seeds were planted in pots filled with moist vermiculite in a greenhouse in Beijing, China. For each plant, seedlings with five replicates were evaluated for ANT and CBB severity. Seedlings at the unifoliate growth stage were inoculated with a 1.2 × 10^6^ spores/ml suspension of *Colletotrichum lindemuthianum* strain 81 using a high-pressure sprayer to evaluate ANT resistance at 20–22°C, 95–100% controlled humidity in a control growth chamber (Balardin et al., 1990). Symptoms were scored at 7 days after inoculation. A 0–9 scale was used for disease severity ratings, with 0 = no symptoms, 1 = a few, very small lesions, 3 = <10%, 5 = 10–25%, 7 = 25–50%, and 9 = more than 50% of inoculated areas showing symptoms. The plants with fully expanded first trifoliate leaves were inoculated by the needle method (Zapata, 2006) to evaluate CBB severity. The fresh inoculum was prepared with *Xanthomonas axonopodis* pv. *phaseoli* isolate XS_2_ at a concentration of 10^8^ CFU/ml. The inoculated plants were maintained in a greenhouse at 25–28°C and rated for CBB infection at 14 days after inoculation using a 1–10 scale (Zapata, 2006) to describe disease symptoms.

### Common bean genome annotation resources

Overall, 27,197 protein-coding genes of the common bean were used (Schmutz et al., 2014) (https://phytozome.jgi.doe.gov/pz/portal.html#!info?alias=Org_Pvulgaris). Transcript data were obtained from the Phytozome database (https://phytozome.jgi.doe.gov/phytomine/template.do?name=One_Gene_Expression&scope=global).

### Identification of sequences encoding NBS motifs in the common bean genome

Predicted proteins from the common bean genome were screened with HMMER V.3 (Finn et al., 2015) using the raw Hidden Markov Model (HMM) corresponding to the Pfam NBS (NB-ARC) family (PF00931). Then, the results were confirmed using the NCBI conserved domain database (http://www.ncbi.nlm.nih.gov/Structure/cdd/wrpsb.cgi) and Pfam database (http://pfam.sanger.ac.uk/). We used Pfam protein families (http://pfam.sanger.ac.uk/) to determine whether these genes encoded TIR (PF01582) or LRR (PF00560, PF07723, PF07725, PF12779, PF13306, PF13516, PF13855, PF14580, PF03382, PF01030, and PF05725) motifs. The COILS program was used to predict the CC motifs at a threshold of 0.9 (http://www.ch.embnet.org/software/COILS_form.html). All “NBS-LRR” tag proteins were downloaded from NCBI to identify partial NBS-LRR genes. The proteins were BLAST searched against this database using an e-value <10^−40^.

### Data analyses

Softberry was used to detect subcellular localization (http://linux1.softberry.com/). The basic information of NBS genes was determined using ExPASy (http://www.expasy.ch/tools/pi_tool.html). The NetNGlyc 1.0 server was used to predict the glycosylation sites of the NBS genes (http://www.cbs.dtu.dk/services/NetNGlyc/). The exon/intron organization of the NBS gene was visualized with the Gene Structure Display Server (GSDS) program (Guo et al., 2007). The upstream promoter sequences of the NBS-LRR genes were identified using the PlantCARE database (Rombauts et al., 1999). Motifs of the NBS proteins were searched on MEME web (Bailey et al., 2009). A phylogenetic tree was constructed in ClustalX (Larkin et al., 2007) based on the amino acid sequences of NBS genes using default parameters, and the tree was generated by the neighbor-joining (NJ) method with bootstrap values using MEGA 4 (Tamura et al., 2007). The heat map was viewed by MeV software (http://www.tm4.org/mev.html).

### Development of SSR markers

SSR markers were developed using the software SSR Locator (da Maia et al., 2008). Various perfect di-, tri-, tetra-, and penta-nucleotide SSR motifs were searched. The numbers of replications of di-, tri-, tetra-, and penta-nucleotide SSR motifs were at least 10, 7, 5, and 4, respectively. The parameters for designing the primers were according to the description by Chen et al. (2014).

### Genotyping

Genomic DNA was isolated from young leaves using a modified cetyltrimethyl ammonium bromide method (Afanador et al., 1993). In total, 283 NBS-SSR markers named with the prefix NSSR were mined based on 68 NBS-coding genes in the common bean. The PCR amplifications were performed in 15-μl reactions containing 20 ng of template DNA, 0.2 μmol l^−1^ primers (Invitrogen, USA), 0.25 mmol l^−1^ dNTPs, 1.5 μl of 10 × Taq buffer with 1.5 mmol l^−1^ Mg^2+^, and 1 U of Taq DNA polymerase in a T100™ Thermal Cycler (Bio-Rad Research, USA). The PCR thermocycling conditions were 5 min at 95°C; followed by 35 cycles of 95°C for 45 s, 53°C for 45 s and 72°C for 45 s; and a final extension of 10 min at 72°C. The PCR products were separated on 8% non-denatured polyacrylamide gels.

### Statistical analysis

Association analysis between markers and phenotypes was performed with TASSEL 2.1 software under the general linear model (Bradbury et al., 2007). Markers were deemed to be associated with ANT or CBB disease if the markers were significant at *p* < 0.01. Population structure was analyzed using the program Structure 2.2 (Pritchard et al., 2000). The parameter of subpopulations (K) was set from 1 to 10 based on 10 independent runs of 100,000 interactions for the length of burn-in period and 100,000 Markov Chain Monte Carlo replications.

### Quantitative real-time PCR (qRT-PCR) analysis

Leaf samples at 0, 6, 12, and 24 h after inoculation were obtained from five seedlings and quickly frozen in liquid nitrogen for extracting RNA. Total RNA was extracted from leaf samples using RNAprep Pure Plant Kit (Tiangen, Beijng) following the manufacturer's instructions. The RNA samples was used to synthesize cDNA by reverse transcription using Transcriptor First Strand cDNA Synthesis Kit according to the manufacturer's manual (Roche, USA). The qRT-PCR reactions were performed on an ABI7500 Real-Time PCR (Bio-Rad Corporation, USA) using *TransStart* Top Green qPCR SuperMix (TransGen Biotech, China). Primers specific for all NBS genes were used for qRT-PCR (Table S3), with normalization to the internal control gene, *Actin* (Borges et al., 2012). Three replicates were performed, and the expression changes were calculated using the 2^−ΔΔCt^ method for each sample (Livak and Schmittgen, 2001). In this study, a five-fold change in expression level was used as a cut-off point. Higher differentially expressed gene levels in susceptible variety samples than in resistant variety samples were denoted by “up-regulated” vs. “down-regulated.”

## Results

### NBS genes in common bean

Overall, 325 gene candidates in which the HMM corresponded to the Pfam NBS family were identified (Table S4). Finally, 227 non-redundant NBS genes were identified, of which 178 full-length protein sequences were used for the subsequent analyses (Table S5) and named PvNBS1-PvNBS178. Furthermore, we obtained 95 R genes without the NBS domain (Table 1, Table S6). We classified 178 full-length proteins into the TNL or CNL families based on the protein sequences of each NBS-LRR candidate. The CNL group included 148 full-length NBS-LRR candidates, NCC (40, without CC and LRR), CN (17, without the LRR), CNL (33), and NLCC (58, without CC). The remaining 30 genes belonged to the TNL group: NTIR TN (4 in the NBS domain from the TIR type and 4 in the TIR and NBS domains but not in the LRR domain), TNL (18 in the TIR, NBS, and LRR domains), and NLTIR (4, without TIR domain) (Table 1, Table S5).

**Table 1:** Classification of the NBS-LRR genes in the common bean genome.

**NBS Type**	**Letter code**	**Common bean**
CNL type		148
NBS	N_CC_	40
CC-NBS	CN	17
CC-NBS-LRR	CNL	33
NBS-LRR	NL_CC_	58
TNL type		30
NBS	N_TIR_	4
TIR-NBS	TN	4
TIR-NBS-LRR	TNL	18
NBS-LRR	NL_TIR_	4
Partial (with NBS)	P_NBS_	50
Partial (without NBS)	P	95

**Figure 1:**
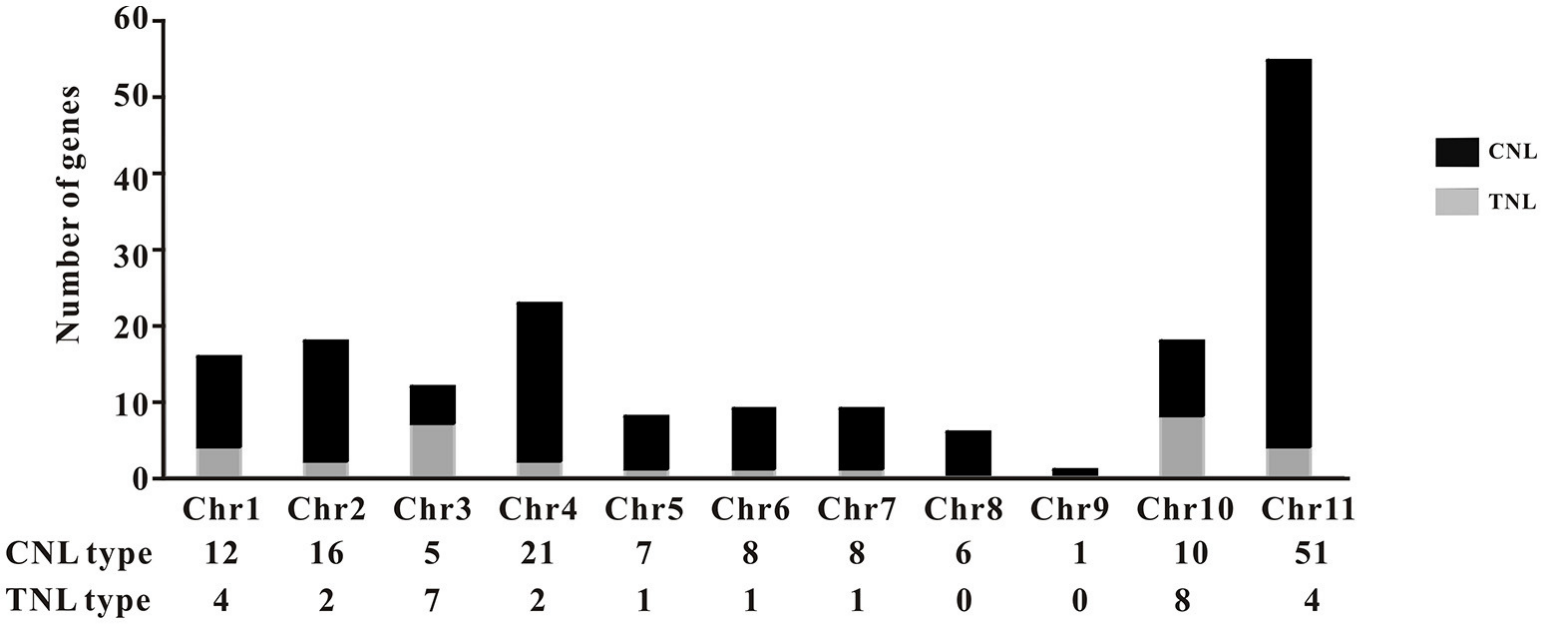
Distribution of the TNL- and CNL-type genes. The distribution of the genes in each type is shown on the common bean chromosomes. The bars are divided into CNL types (black) and TNL types (gray).

**Figure 2:**
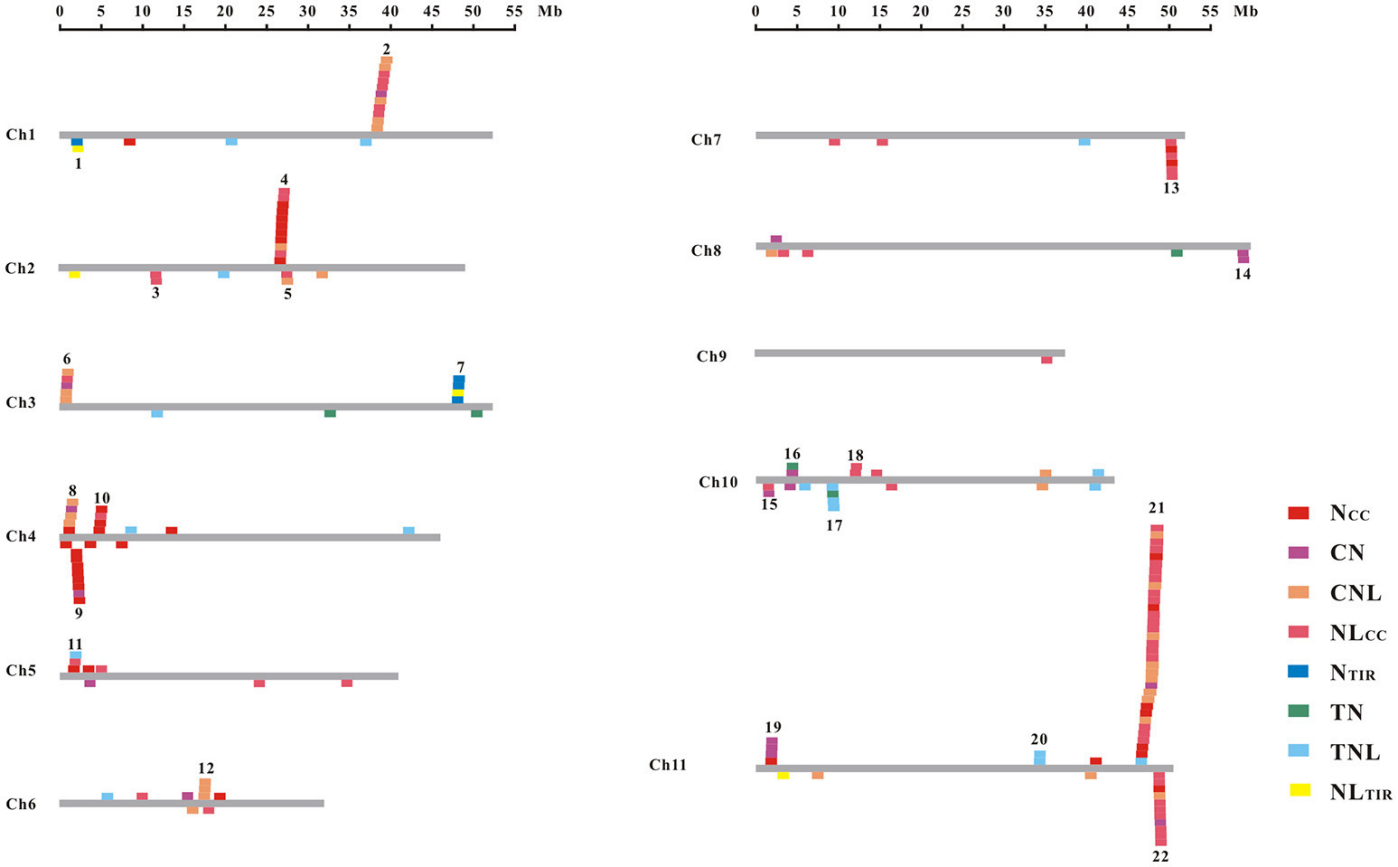
Chromosomal distribution of the NBS-LRR genes in the common bean. The gray bars represent all 11 chromosomes in the common bean. Boxes across each bar designate the location of each gene. The cluster number is shown at the top of each cluster.

The common bean NBS-LRR genes are distributed across all 11 chromosomes (Chr1-Chr11); however, *Phvul.L001800*, *Phvul.L003300*, and *Phvul.L006800* remained on unmapped scaffolds (Figure 1, Table S7). The PvNBS genes distribution on common bean chromosomes have no any rules. Total of 96 PvNBS genes were located on three chromosomes: 23 PvNBS genes on Chr4, 18 PvNBS genes on Chr10, and 55 PvNBS genes on Chr11. CNLs were present on Chr1-Chr11, whereas TNLs were absent from Chr8 and Chr9. The greatest number of NB-LRRs was identified on Chr11, which harbored 55 genes; however, only one NBS-LRR gene was distributed on Chr9. Moreover, 39% of all the TNL genes were distributed on Chr10, whereas 28% of all the CNL genes were distributed on Chr11. The distribution of NBS-LRR genes tended to form clusters among the chromosomes. To form a cluster, there must be > 200 kb distance between neighboring NBS-LRRs and fewer than eight non-NBS-LRR genes between the NBS-LRRs (Lozano et al., 2015). Based on this standard, we identified 129 NBS-LRR genes belonging to 22 clusters, and the average number of NBS-LRR genes in a cluster was 5.86 (Figure 2, Table S7). Chromosomes 10 and 11 had the highest number of clusters at 4. The number of members per cluster ranged from 2 to 34. Cluster 21 had the highest number of members at 34 genes. In addition, 49 NBS-LRR singletons were distributed on all the chromosomes. The cluster size ranged from 9,264 to 1,805,165 bp.

### Gene structural characteristics, phylogenetic relationships, and analysis of the conserved motifs of the NBS genes

We analyzed the genomic sequences, CDS, protein lengths, NW, pI, glycosylation sites, and subcellular localization of the 178 full-length NBS genes (Table S5). The length of the genomic sequences of these NBS genes ranged from 1,157 to 15,770 bp, the CDS length ranged from 978 to 9,480 bp. The protein length ranged from 325 AA (*Phvul.010G054400*) to 3,159 AA (*Phvul.010G091100*), and the MW ranged from 37.20 kDa to 360.50 kDa, and the pI ranged from 5.04 to 9.17. We also predicted the number of glycosylation sites, which ranged from 0 to 37. *Phvul.010G091100* contains 37 sites, whereas *Phvul.003G247500* does not contain any sites. The results of subcellular localization prediction showed that 170 proteins were located in the extracellular region, 6 NBS proteins were located in the cytoplasm, *Phvul.008G061300* was located in the plasma membrane, and *Phvul.010G054400* was located in the endoplasmic reticulum. The average number of exons of these NBS genes was 3.10. We also found that the average number of exons in the TNL genes was higher (5.56) than the number of exons in the CNL genes (2.74). Furthermore, 39.4% of the CNL genes had a single exon.

All common bean NBS protein sequences were used to conduct a phylogenetic tree. Overall, 178 NBS genes can be separated to two different groups. The first clade included all CNL-type proteins and could be classified into nine major groups: I, II, III, IV, V, VI, VII, VIII, and VX (Figure S1). Group I was the largest group (49 members) and accounted for 33.1% of all CNL-type proteins, and most of the members were located on Chr11. In contrast, Group III contained only one member, *Phvul.005G031200*. We also noticed that most of members of groups II and V were located on Chr4 and Chr2, respectively. The second clade contained all TNL proteins and showed clear separation with the CNL.

**Figure 3:**
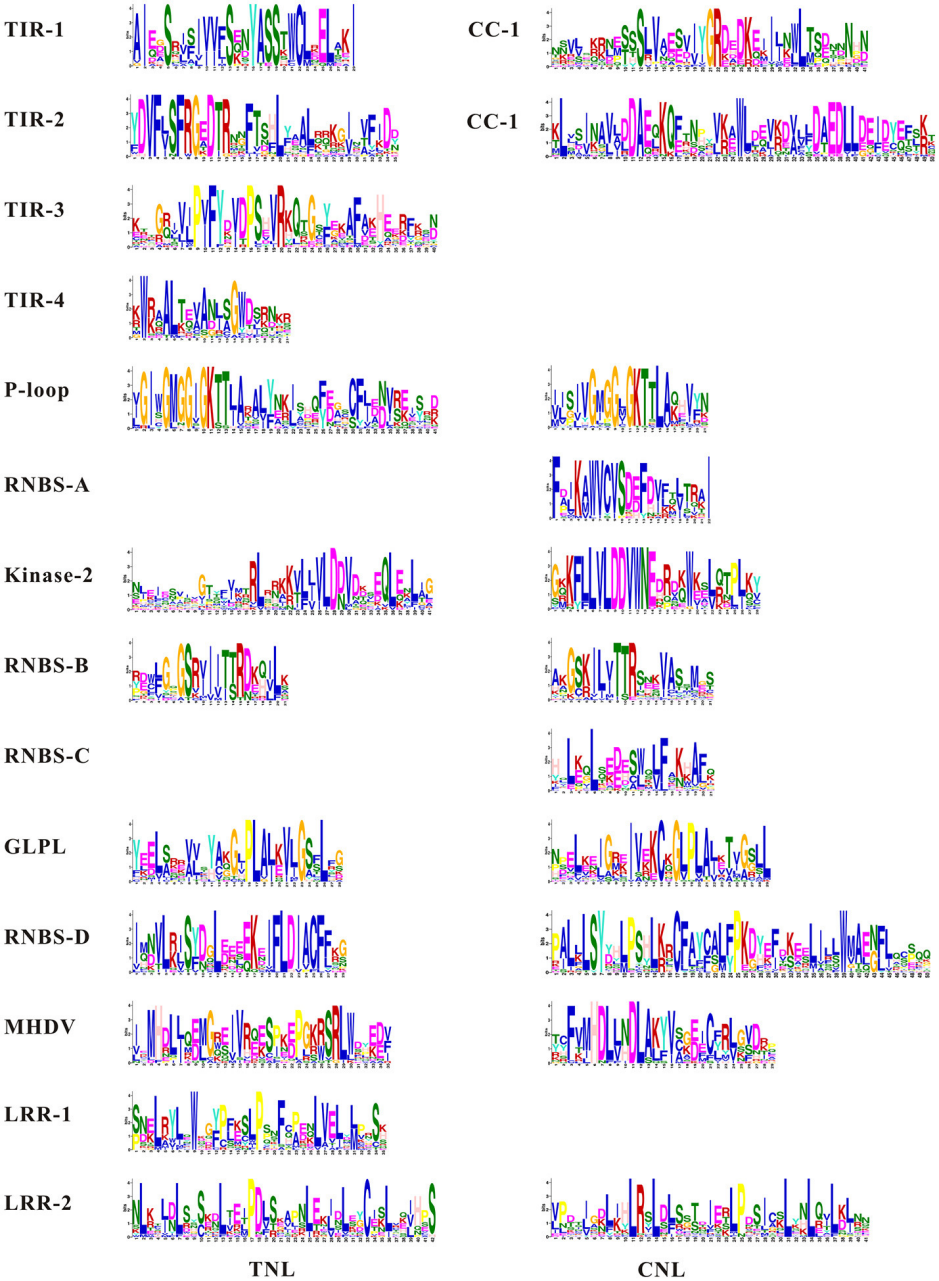
MEME analysis of the TNL and CNL proteins. Different colored letters represent amino acids belonging to the different families.

**Figure 4:**
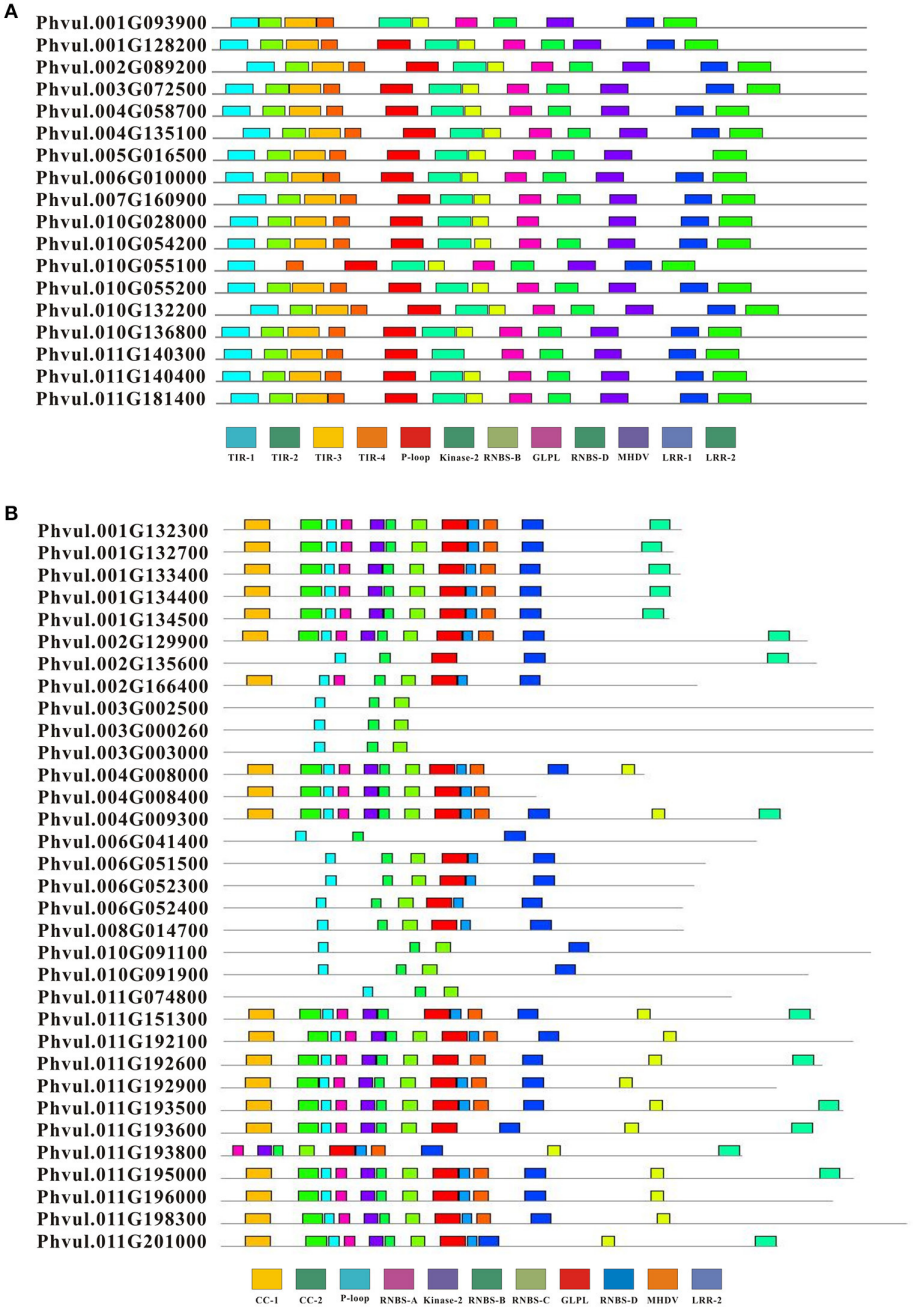
Composition of the conserved motifs in NBS genes in the common bean. The conserved motifs of the common bean NBS-LRR genes were elucidated using MEME. The conserved motifs are represented by different colored boxes. **(A)** The conserved motifs in TIR-NBS-LRR. **(B)** The conserved motifs in CC-NBS-LRR.

We detected conserved motifs with the amino acid sequences of complete CNL (33) and TNL (18) proteins using MEME (Figures 3, 4). The results showed that the P-loop, kinase-2, NBS-A to -D, GLPL, and MHDV motifs were also detected in the CNL members, whereas the NBS-A and -C motifs were absent in the TNL group. Then, the P-loop, kinase-2, GLPL, and MHDV displayed a high level of similarity between the CNL and TNL proteins, and the RNBS-D motif presented low similarity between the common bean CNL and TNL proteins. Furthermore, the MHDV motif in the CNL and TNL proteins in the common bean was modified into a MHDL motif. However, motifs TIR-1, TIR-2, TIR-3, and TIR-4 were detected in the N-terminus of the TNL protein, and the order of these motifs was consistent with other species. Among the TNL proteins, TIR-2 and TIR-4 were deleted in the *Phvul.010G055100* protein, whereas other TNL proteins contained all four motifs. In addition, the CC-1 and CC-2 motifs were detected at the N-terminus of the CNL protein. Half of the CNL proteins contained these two motifs. Finally, we detected two motifs and one motif in the LRR region of the TNL and CNL proteins, respectively.

### Cis-element analysis

Approximately 1,500 bp sequences upstream of the start codon were isolated and used to identify putative CAREs using the PlantCARE database. Ninety similar CAREs associated with abiotic stress, hormones, tissue-specific expression, light responsiveness, and other elements were identified (Table S8). A large number of light-responsive elements were detected in the promoter regions of the PvNBS genes, such as Box 4, Box I, G-box, and the GAG-motif. Furthermore, CAREs involved in plant hormones were also identified, for example, abscisic acid responsiveness element (ABRE, CE1 and CE3), the gibberellin-responsive element (TATC-box and P box), and salicylic acid responsiveness element (CGTCA-motif and SARE). Finally, elements that are important in abiotic stress were identified in most of the PvNBS genes, such as the WUN-motif and Box-W1, were detected. Moreover, TC-rich repeats involved in defense and stress responsiveness. In addition, G-box, GT1, and Box-W1, which are involved in defense gene expression induced by pathogen or fungal elicitors, were also detected in most of the NBS-LRR genes (Rushton et al., 1996; Faktor et al., 1997; Yamamoto et al., 2004; Shi et al., 2013).

### Expression profiles of the NBS genes in response to anthracnose and common bacterial blight

Expression data were not available for *Phvul.007G254400, Phvul.004G053000*, or *Phvul.010G025700* in the publicly RNA-seq data. The expression profiles of 175 NBS-LRR genes were obtained in 9 common bean tissues (Figure S2, Table S9). Most of the PvNBS genes were expressed in 9 tissues (120 genes, 68.6%), and no tissue expressed all the PvNBS genes. PvNBS genes were most abundant in the stems (161 genes, 92.0%), followed by the roots (160 genes, 91.4%). Few PvNBS genes were expressed in the flower buds (142 genes, 81.1%). All the PvNBS genes were divided into 10 groups based on their expression patterns. *Phvul.001G018800, Phvul.003G247200, Phvul.008G014700*, and *Phvul.011G014300* were showed highly expressed in most of common bean organs throughout the growing stage, whereas *Phvul.001G132700, Phvul.001G132800, Phvul.002G129500, Phvul.002G130300, Phvul.005G020300, Phvul.010G023200, Phvul.011G154000, Phvul.011G181600*, and *Phvul.011G194900* were expressed at low levels in the common bean. In addition, *Phvul.007G254200, Phvul.010G055100, Phvul.010G025100*, and *Phvul.011G193800* were specifically expressed in the roots, young pods, flowers, and flower buds, respectively.

Next, the expression profiles of all PvNBS genes in response to ANT and CBB infection in resistant and susceptible lines were studied by qRT-PCR. Among 178 NBS genes, 7 genes (*Phvul.002G135100, Phvul.004G005600, Phvul.004G008000, Phvul.004G015800, Phvul.005G020300, Phvul.011G192600*, and *Phvul.011G196100*) were not analyzed by qRT-PCR as no suitable primers were screened.

Of the remaining 171 NBS genes, 67 genes showed differential expression levels (fold change ≥5 or ≤0.2) between resistant and susceptible genotypes for ANT and CBB (Table S10). For ANT, the number of up-regulated genes (48) was higher than the down-regulated genes (19) in the susceptible cultivar (Jingdou) compared to the resistant cultivar (Hongyundou). By contrast, more down-regulated genes (43) than up-regulated genes (24) were identified in the susceptible line (Bilucaidou) compared to the resistant line (HR45) for CBB. In addition, 29 genes showed differential expression in resistant and susceptible cultivars for both diseases. Meanwhile, 11 genes showed the same expression pattern in resistant and susceptible genotypes for two diseases: 4 genes were up-regulated, and 7 genes were down-regulated. Eighteen genes shared opposite tendency of expression changes between ANT and CBB: 14 genes were up-regulated for ANT but down-regulated for CBB, and 4 genes were down-regulated for ANT but up-regulated for CBB (Figure S3, Table S10).

According to ANT, 113 NBS genes showed differential expression (fold change ≥5 or ≤0.2) in at least at one time point compared to 0 h (Table S10). A range of expression patterns of these genes was observed in specific genotypes at different time points: 40 genes showed significant responses only in the resistant genotype (Hongyundou) and 37 genes only in the susceptible genotype (Jingdou) following fungal infection, whereas 36 genes were expressed in both genotypes after inoculation. Among the 36 genes, 31 showed differential expression patterns in the resistant and susceptible genotypes for ANT from 0 to 48 h after inoculation: 12 genes showed up-regulation only in the resistant genotype at 6, 12, or 24 h but down-regulation or no change in the susceptible genotype at any time point. In contrast, 5 genes showed up-regulation only in the susceptible genotype but down-regulation or no regulation in the resistant genotype at 6 h.

For CBB, the expression of 141 genes was significantly altered (fold change ≥5 or ≤0.2) in at least at one time point compared to 0 h (Table S10). Among these genes, 40 genes were changed only in the resistant genotype (HR45), 49 genes only in the susceptible (Bilucaidou), and 52 genes in both genotypes induced by XS_2_. Of the common 52 genes, 10 genes showed the same expression pattern in two genotypes from 0 to 48 h after inoculation, 11 genes showed down-regulation only in the resistant genotype but up-regulation or no regulation in the sensible cultivar after pathogen infection, and 8 genes showed higher expression levels in the resistant genotype than in the susceptible genotype after inoculation. Twelve genes in the resistant genotype were increased to the highest expression levels at 12 h only and then decreased at 24 h, while these genes remained stable at 6 and 12 h or reached lowest expression levels at 12 h then increased at 24 h in the susceptible genotype.

### Development of SRR markers around the NBS genes

We developed SSR markers around the NBS-LRR genes to improve the accuracy of NBS gene mapping. For the singletons, 500-kb sequences upstream and downstream of the gene were isolated and used to detect the SSR locus (Table S11). For the clusters, the sequences between the first and last genes were isolated and used to detect the SSR locus (Table S12). First, we searched for SSR motifs in the DNA sequences. Overall, 245 and 1,149 perfect microsatellites were detected in the DNA sequences of the NBS-LRR genes and singletons, respectively. In total, 571 SSR markers were developed for the NBS genes (Tables S11, S12). The microsatellites of these SSR markers included 369 (64.6%) di-nucleotide motifs, 107 (18.7%) tri-nucleotide motifs, 41 (7.2%) tetra-nucleotide motifs, and 54 (9.5%) penta-nucleotide motifs. The AT/TA types were more abundant (86.7%) than the AG/TC (8.7%) and AC/TG types (0.8%). However, we don't identified CG/GC types in the DNA sequences of the NBS-LRR regions.

### Association studies of ANL and CBB resistance genes using NBS-SRR markers in the common bean

To identify genes related to ANL and CBB resistance in the common bean, we chose 68 markers for association studies, with one marker for each NBS singleton/cluster. The results of the structure simulation showed that a maximum log likelihood was attained at *K* = 2, suggesting that two subpopulations could contain all accessions with the greatest probability (Figure S4). Finally, nine markers were associated with ANT and seven markers with CBB disease as indicated by the probability values (*p* < 0.01) (Table 2). The phenotypic variance explained (*R*^2^) ranged from 8.9 to 18.0% for ANT and 6.0 to 24.4% for CBB. Statistically significant associations involving NBS-SSR markers were observed for ANT on seven chromosomes and for CBB on five chromosomes. On chromosomes for ANT, two SSR markers located on Chr11, NSSR271 and NSSR281, and the marker NSSR24 (Chr2) showed significant (*p* < 0.001) associations with ANT resistance. The marker NSSR271 was the most significantly associated with ANT, explaining 18.0% of the phenotypic variation. On chromosomes for CBB, four SSR markers, NSSR32 and NSSR34, both located on Chr3, and NSSR240 (Chr4) and NSSR245 (Chr7) showed significant (*p* < 0.001) associations. NSSR240 showed the most significant association with CBB, explaining 24.4% of the phenotypic variation. The SSR marker NSSR65, located on Chr4, was associated with both diseases, suggesting a possible pleiotropic effect.

**Table 2:** SSR markers associated with ANT and CBB severity in 183 common bean germplasms (*p* < 0.01).

**Marker**	**Gene**	**Marker position (bp)^a^**	**Chromosome**	**ANT**			**CBB**		
				***F* ratio**	***p*–value**	***R*^2^ (%)^b^**	***F* ratio**	***p* value**	***R*^2^ (%)^b^**
NSSR8	*Phvul.001G128200.1*	36182090–36174911	1	2.89	4.8 × 10^−3^	11.7			
NSSR24	*Phvul.002G166400.1*	30830902–30828082	2	8.91	2.0 × 10^−4^	8.9			
NSSR32	*Phvul.003G072500.1*	10994765–11000821	3				4.91	3.3 × 10^−4^	13.2
NSSR34	*Phvul.003G129700.1*	31823313–31828331	3				4.02	8.6 × 10^−4^	12.4
NSSR65	*Phvul.004G135100.1*	41368421–41363361	4	3.51	1.5 × 10^−3^	12.0	3.01	5.3 × 10^−3^	11.0
NSSR73	*Phvul.005G020300.1*	1746532–1749452	5	6.61	1.7 × 10^−3^	6.6			
NSSR117	*Phvul.006G066800.1*	18546221–18542599	6	4.37	2.2 × 10^−3^	9.4			
NSSR197	*Phvul.011G030000.1*	2539794–2544217	11				5.66	4.2 × 10^−3^	6.0
NSSR234	Cluster 8	673367–877733	4	3.65	3.6 × 10^−3^	9.0			
NSSR240	Cluster 10	4020210–4069578	4				2.66	4.9 × 10^−4^	24.4
NSSR245	Cluster 13	49260558–49310456	7				5.54	9.3 × 10^−5^	13.7
NSSR260	Cluster 16	3728084–3754731	10				4.02	8.5 × 10^−3^	6.4
NSSR265	Cluster 17	8568461–8788327	10	3.52	1.5 × 10^−3^	12.2			
NSSR271	Cluster 19	1111792–1156724	11	4.73	3.0 × 10^−5^	18.0			
NSSR281	Cluster 21	45753810–47558975	11	4.93	8.7 × 10^−4^	9.7			

a *Physical location of genes in the G19833 genome*.

b *R^2^ indicates the percent of phenotypic variation explained by each marker*.

Furthermore, we compared these loci with known ANT and CBB loci. Among these association markers, NSSR8, NSSR65, NSSR117, NSSR234, NSSR271, and NSSR281 may be located at the same regions identified for ANT resistance in previous studies (Ferreira et al., 2013; Campa et al., 2014; Gonzalez et al., 2015; Perseguini et al., 2016; Zuiderveen et al., 2016). Among the CBB resistance loci, NSSR32, NSSR34, NSSR240, and NSSR245 may be located in the same CBB resistance QTL region based on map positions reported in previous studies (Miklas et al., 2006; Liu et al., 2008; McConnell et al., 2010; Shi et al., 2011). The NSSR65 marker was associated with both diseases but was located far away from the other marker linked with the CBB resistance QTL. In addition, one important locus (SAP6-QTL) for CBB resistance in the common bean has been reported at approximately 39.9 Mb on Chr10 (Miklas et al., 2003; Shi et al., 2011). NSSR260, which was associated with CBB resistance, was identified at 3.7 Mb on Chr10. Previous studies have associated different regions of Chr10 with CBB resistance. No major ANT resistance genes have been reported on Chr10, except a SNP at 3.8 Mb associated with a minor QTL (Zuiderveen et al., 2016). The NBS-SSR marker NSSR265 at 8.6 Mb on Chr10 identified in our study was associated with ANT resistance, potentially revealing a new region for ANT resistance. Furthermore, NSSR24 (Chr2) and NSSR73 (Chr5) are located far from other markers linked with ANT resistance genes in previous studies based on physical position (Perseguini et al., 2016; Zuiderveen et al., 2016), suggesting new locations for ANT resistance.

For these new locations, NSSR24, NSSR65, NSSR73, NSSR260, and NSSR265, we selected another nature population for association studies of ANT and CBB resistance (Table S13). The results showed that only NSSR65 for CBB was not detected in the population by the probability values (*p* < 0.01). In the population, NSSR260 explained 14.9% of the phenotypic variation for CBB. NSSR24, NSSR73, and NSSR265 explained 9.8, 8.8, and 15.9% of the phenotypic variance for ANT, respectively. The result confirmed the utility of these markers associated with these new loci linked to ANT/CBB.

Furthermore, we analyzed the expression patterns of the five locations, including nine NBS genes in resistant and susceptible cultivars for ANT/CBB. Among these NBS genes, two belonged to cluster 16 co-localized with a locus for CBB resistance, and four belonged to cluster 17 co-localized with a locus for ANT resistance (Table 2, Table S7). Of these NBS genes, *Phvul.010G054400* and *Phvul.010G055200* showed down-regulation only in the resistance line (Hongyundou) at all times points after inoculation with the ANT pathogen, race 81. In terms of the levels of expression, *Phvul.010G054400* and *Phvul.010G055100* were expressed approximately 21- and 43-fold higher in Hongyundou than in Jingdou, respectively. For CBB, *Phvul.010G025200* expression in the resistant cultivar (HR45) was significantly lower than in the susceptible cultivar (Bilucaidou) before inoculation. The expression level of the gene increased significantly at 0–12 h and was slightly reduced at 24 h in the resistant genotype after infection. In the susceptible genotype, expression increased at 0–6 h then dropped significantly to almost no noticeable expression at 12 h, followed by significantly increased expression at 24 h but was still significantly lower than that in HR45.

## Discussion

The common bean is the world's most important food legume and belongs to the Fabaceae family, which includes many economically species, for example, mung bean, fava bean, and cowpea (Gepts et al., 2005; Cannon et al., 2009; Blair et al., 2016). The common bean is important as a source of grain for human consumption, and it is also used to produce protein concentrates for animal feed (Jansman, 2005). Because of the importance of the common bean, the development of disease-resistant cultivars is essential. Thus, the common bean genome sequences provides us an opportunity to identify, classify, and develop NBS-SSR markers for molecular breeding of disease-resistant cultivars.

Up to now, many important NBS-LRR genes involving to disease resistance have been cloned (Bryan et al., 2000; Zhou et al., 2001; Periyannan et al., 2013; Saintenac et al., 2013). In this article, 228 NBS domain genes and 95 partial NBS-LRR genes were detected, which represented 1.19% of all the common bean's predicted proteins. This number is higher than that of cassava (~0.9%), *Arabidopsis* (~0.43%), and *V. vinifera* (~0.91%) (Meyers et al., 2003; Yang et al., 2008; Lozano et al., 2015). NBS genes can be classified into the TNL or CNL families. The TIR and CC sequences have obvious differences between dicots and monocots (Cheng et al., 2012). The TIR motif is rarely present in disease resistance genes in monocots, such as *O. sativa, T. aestivum, Z. mays, S. bicolor, B. distachyon*, and other plants (Zhou et al., 2004; O'Toole et al., 2008; Cheng et al., 2012; Tan and Wu, 2012; Jain et al., 2013; Ling et al., 2013; Bouktila et al., 2014; Mace et al., 2014; Wang et al., 2014; Gu et al., 2015; Singh et al., 2015). In contrast, most dicots contain TNL, including *A. thaliana* (51), *P. trichocarpa* (119), *M. truncatula* (152), *M. x domestica* (218), and *G. raimondii* (35) (Meyers et al., 2003; Lurin et al., 2004; Shiu et al., 2004; Arya et al., 2014; Song and Nan, 2014; Yu et al., 2014). Among these NBS-LRR genes, we identified 18 TIR-NBS-LRRs and 33 CC-NBS-LRRs. The number of CNL genes was greater than the number of TNL genes, which is generally consistent with the results observed in other dicots, such as *V. vinifera* (203 CNL and 97 TNL), *M. esculenta* (117 CNL and 29 TNL), and *S. lycopersicum* (118 CNL and 18 TNL) (Yang et al., 2008; Lozano et al., 2012; Andolfo et al., 2014).

NBS-LRR genes contain three domains: an N-terminal TIR/CC domain, a central NBS domain, and a C-terminal LRR domain (Meyers et al., 2003; Song and Nan, 2014). TIR-1, -2, -3, and -4 were detected in the N-terminus of TNL genes from the common bean, which is consistent with results observed in *Arabidopsis thaliana* and *Populus trichocarpa* (Meyers et al., 2003; Kohler et al., 2008). In contrast, only two motifs were identified in the N-terminus of CNL genes from the common bean, which is inconsistent with the results observed in other species, such as *A. thaliana* or *P. trichocarpa* (Meyers et al., 2003; Kohler et al., 2008). For the C-terminal LRR domain, two and one motifs were detected in the TNL and CNL families, respectively. The NBS-encoding genes are divided into eight conserved motifs (Meyers et al., 2003). RNBS-A and RNBS-C were deleted in the TNL family from the common bean; however, the NBS-A motif was also deleted in the TNL members from *Brassica rapa* (Mun et al., 2009). The sequences of the motifs also differed in other species. Furthermore, the MHDV motif in TNL and CNL proteins was often changed to a MHDL motif, which is different from that of *B. rapa* (MHSL), *P. trichocarpa* (MHDL, QHDV and QHDL), and maize (MHDL) (Kohler et al., 2008; Mun et al., 2009; Cheng et al., 2012).

As previously reported, the complexity of the clusters of NBS genes caused rapid gene evolution (Friedman and Baker, 2007). These clusters have been identified in many plants. Seventy percent of the cassava NBS-LRR genes are located within a cluster (Lozano et al., 2015). The NBS genes of *Solanum tuberosum* were identified in high-density clusters (Lozano et al., 2012). In soybean, 40 NBS-LRR genes were clustered on Chr16, and 32 NBS-LRR genes were clustered on Chr18 (Kang et al., 2012). In *Brachypodium distachyon*, 43 genes were located in 11 clusters (Tan and Wu, 2012). In *M. truncatula*, 309 NBS genes were located in 78 gene clusters (Song and Nan, 2014). In the common bean, 26 CNL-encoding genes were found in the approximately 650-kb region (David et al., 2009). In this study, the average number of NBS proteins in a cluster was 5.86, and it was greater than the ratios in *A. thaliana* (3.21), maize (2.77), *B. distachyon* (4) and *M. truncatula* (3.96) (Meyers et al., 2003; Cheng et al., 2012; Tan and Wu, 2012; Song and Nan, 2014).

In this paper, we analyzed the expression patterns of these NBS genes at different organs and different times after ANT/CBB pathogen infection. Expression pattern studies are the foundation of expounding gene function. RNA-seq data showed that some NBS genes were expressed in all organs examined. These results are similar to NBS genes from other crops, such as *Pi64* in rice (Ma et al., 2015), *Sr35* in wheat (Saintenac et al., 2013), and *ZmNBS28* in maize (Cheng et al., 2012). In contrast, some NBS genes were specifically expressed at only one organ or stage, i.e., *Phvul.007G254200* was expressed in the root, *Phvul.010G055100* in the young pod, and *Phvul.010G025100* in the flower. Similar reports are available in other crop studies. For example, *Pb1* showed resistance only during adult stages in rice (Hayashi et al., 2010), *Glyma12g03040* was expressed at 3-week-old nodules in soybean (Hayashi et al., 2012), and *ZmNBS1* was expressed at the root in maize (Cheng et al., 2012). Some NBS genes showed significant expression differences between resistant and susceptible lines, such as *Phvul.004G008400* and *Phvul.011G192400* for ANT disease and *Phvul.002G129900* and *Phvul.011G200900* for CBB disease. Differences between genotypes were also observed in other crops, such as *Sr35* in wheat (Saintenac et al., 2013), *Bol016084* in cabbage (Kim et al., 2015), and *Phvul.001G243700* in the common bean (Chen et al., 2017). In addition, some genes were found to be highly induced in resistant lines after pathogen infection. For instance, *Phvul.004G012900, Phvul.004G013100, Phvul.001G134500*, and *Phvul.002G129900* expression was increased only in the resistant line after inoculation with pathogens. The *Arabidopsis* gene *AT3G14460* was homologous to *Phvul.001G134500*, which was up-regulated 7.3-fold in *Arabidopsis thaliana* post-*Bacillus subtilis* FB17 colonization (Lakshmanan et al., 2013). In contrast, some gene expression changed extremely in susceptible cultivars infected with pathogen, including *Phvul.001G132800* and *Phvul.004G016500* for ANT disease and *Phvul.004G036300* and *Phvul.006G051500* for CBB disease. However, all the above expression data provide valuable information for further studies of ANT/CBB.

Extensive discovery and identification of disease resistance genes are the basis of resistance breeding, and SSR markers are widely used to map the disease resistance genes in the common bean. Molecular markers linked with QTLs/genes for important traits have been routinely developed for several crops, such as rice and wheat (Virk et al., 1996; Maccaferri et al., 2005; Breseghello and Sorrells, 2006). In the present study, we developed a series of SSR markers based on the sequence near the NBS genes. These NBS-SSR markers are closely linked to the NBS genes and display almost no recombination; thus, they can be used as co-segregation markers. These NBS-SSR markers offer a powerful tool for the identification of resistant genotypes. In this study, we identified ANT and CBB resistance genes using these NBS-SSR markers. Nine NBS-SSR markers showed significant associations with ANT resistance and seven with CBB resistance (Table 2). Among these loci, three markers (NSSR24, NSSR73, and NSSR265) associated with ANT resistance and two markers (NSSR65 and NSSR260) associated with CBB resistance may be new locations by comparing to previous studies (Liu et al., 2008; Shi et al., 2011; Ferreira et al., 2013; Gonzalez et al., 2015; Perseguini et al., 2016; Zuiderveen et al., 2016). In this study, NSSR24, NSSR65, NSSR73, NSSR260 and NSSR265 were examined in a new nature population; it was confirmed that NSSR24, NSSR73, and NSSR265 also associated with ANT resistance, and NSSR260 associated with CBB resistance in the new population. Furthermore, we obtained the expression data of the genes only in NSSR24, NSSR65, NSSR260, and NSSR265 locations because no suitable primers were designed for the genes in NSSR73. The gene expression in NSSR24 and NSSR65 was not changed between resistant and susceptible lines or before/after inoculation. In contrast, *Phvul.010G054400*, one of four genes in the NSSR265 location, was expressed higher in the resistance genotype than in the susceptible genotype before inoculation. However, the gene expression for ANT declined sharply early after infection in the resistance genotype, whereas there was no change in the susceptible genotype at the cut-off level of 5.0 compared to the control. Therefore, it is possible that *Phvul.010G054400* may act as a negative regulator of ANT resistance at the locus in Hongyundou during fungal infection. In addition, the sequence of the NBS gene *Phvul.010G054400* was highly similar with the *Arabidopsis RPP5* gene (*AT4G16950*). The *RPP5* gene belongs to TIR-NBS-LRR class, which confers higher resistance to soybean rust or other plant diseases (Eckardt, 2007; Lemos et al., 2011). According to NSSR260 for CBB, *Phvul.010G025200*, one of the two genes in the location, was homologous to the *Arabidopsis* gene *AT5G36930*, which encodes a TIR-NBS-LRR disease resistance protein (Tabata et al., 2000). The expression level of *Phvul.010G025200* in the resistant genotype for CBB was higher than in the susceptible genotype after pathogen infection, suggesting that the NBS-LRR gene may increase its expression upon pathogen attack. Therefore, these results together with the genome-wide association study for ANT and CBB provide a useful resource for NBS gene functional characterization and genetic improvement of the common bean, and perhaps for other food legumes.

In the present study, the basic information of NBS-LRR members were evaluated in the common bean. We also performed expression pattern in the different common bean organs and in response to ANT and CBB. It is important that we obtained and validated three and one new NBS loci associated with ANT and CBB, respectively. All these results will accumulate our knowledge about NBS genes in common bean, and provide important data to expound how the PvNBS genes to respond under the disease stress.

## Author contributions

JW and SW conceived and designed the experiments. JW, JZ, and LW performed the experiments. JW and JZ analyzed the data. JW, JZ, and SW contributed to the writing of the manuscript. All authors read and approved the final manuscript.

### Conflict of interest statement

The authors declare that the research was conducted in the absence of any commercial or financial relationships that could be construed as a potential conflict of interest.

## Supplementary material

Figure S1 Phylogenetic tree of NBS genes from the common bean. The tree was constructed in MEGA4.0 using the neighbor-joining method with 1,000 bootstrap replicates. Red dots represent the TNL type. Each protein is encoded as follows: ID number + Domains present (TNL, TN, NTIR, NLTIR, CNL, CN, NCC, NLCC).

Figure S2 Heat map of the expression profiles of the NBS-LRR genes across different tissues. The expression data were downloaded from the Phytozome web site. The color scale shown below the heat map represents the expression values (FPKM), with green indicating low levels, and red indicating high levels of transcript abundance.

Figure S3 Number of differentially expressed genes for ANT and CBB. The numbers of differentially expressed genes that were exclusively up- or down-regulated for one disease are shown in each circle. The numbers of genes with common or opposite tendency of expression changes between different diseases are shown in the overlapping regions. The total numbers of up- or down-regulated genes for each disease are shown outside of the circles.

Figure S4 Estimated population structure of 183 common bean accessions (*K* = 2). Each group (subpopulation) is represented by a different color.

Table S1 The phenotypic data of 183 common bean germplasms for ANT and CBB.

Table S2 The phenotypic data of 100 common bean germplasms for ANT and CBB.

Table S3 The primer sequences of NBS genes for qRT-PCR.

Table S4 List of the 325 NBS proteins in the common bean.

Table S5 List of the 178 putative NBS family genes identified in the common bean.

Table S6 List of the 110 R genes without the NBS domain in the common bean.

Table S7 Positions of the anchored NBS-LRR genes and cluster assignments.

Table S8 Predicted promoter elements of common bean NBS genes.

Table S9 Expression data of NBS genes in the common bean (the source of the data was the phytozome).

Table S10 The relative expression data of NBS genes in resistant and susceptible lines for ANT and CBB at four different time points.

Table S11 SSR loci and markers for singletons.

Table S12 SSR loci and markers for clusters.

Table S13 Validation of SSR markers linked to new loci for ANT and CBB in 100 common bean germplasms (*p* < 0.01).
