# Supplementary figures and images for: Genome-Wide Association Study Identifies NBS-LRR-Encoding Genes Related with Anthracnose and Common Bacterial Blight in the Common Bean
Source: Front Plant Sci. 2017 Aug 9;8:1398. doi: 10.3389/fpls.2017.01398 (PMC5552710; doi:10.3389/fpls.2017.01398)

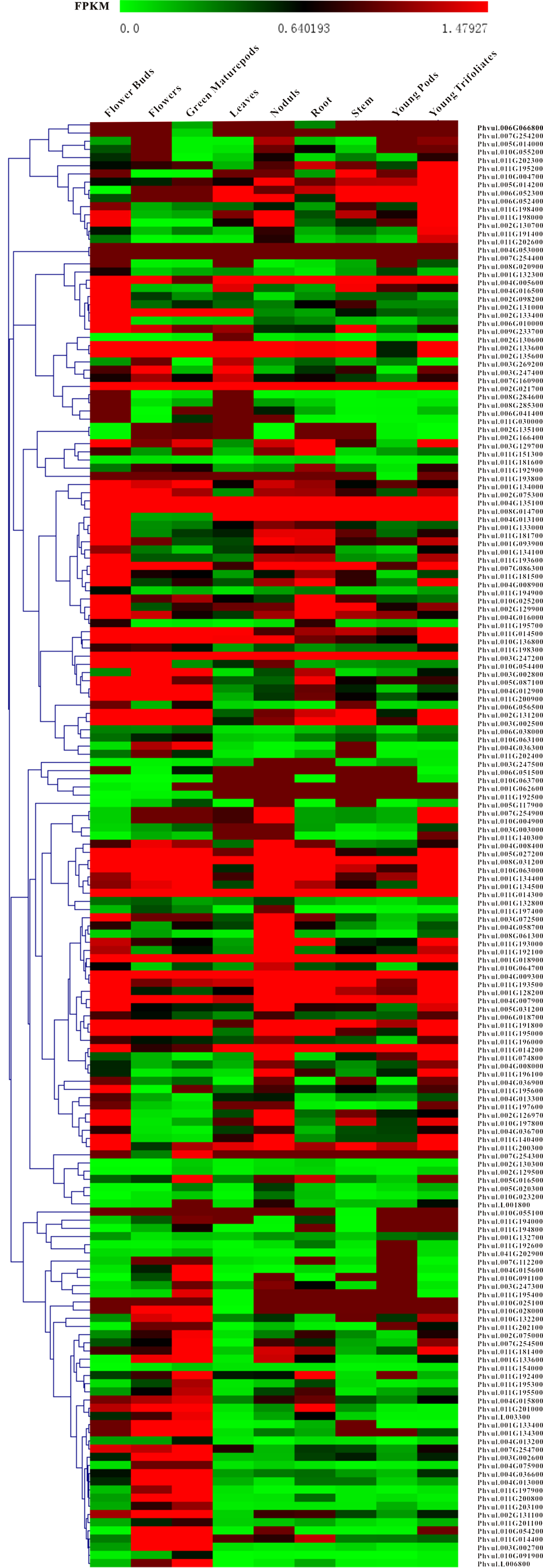

Supplement: Figure S1 — Phylogenetic tree of NBS genes from the common bean. The tree was constructed in MEGA4.0 using the neighbor-joining method with 1,000 bootstrap replicates. Red dots represent the TNL type. Each protein is encoded as follows: ID number + Domains present (TNL, TN, NTIR, NLTIR, CNL, CN, NCC, NLCC). [file Image1.TIF]

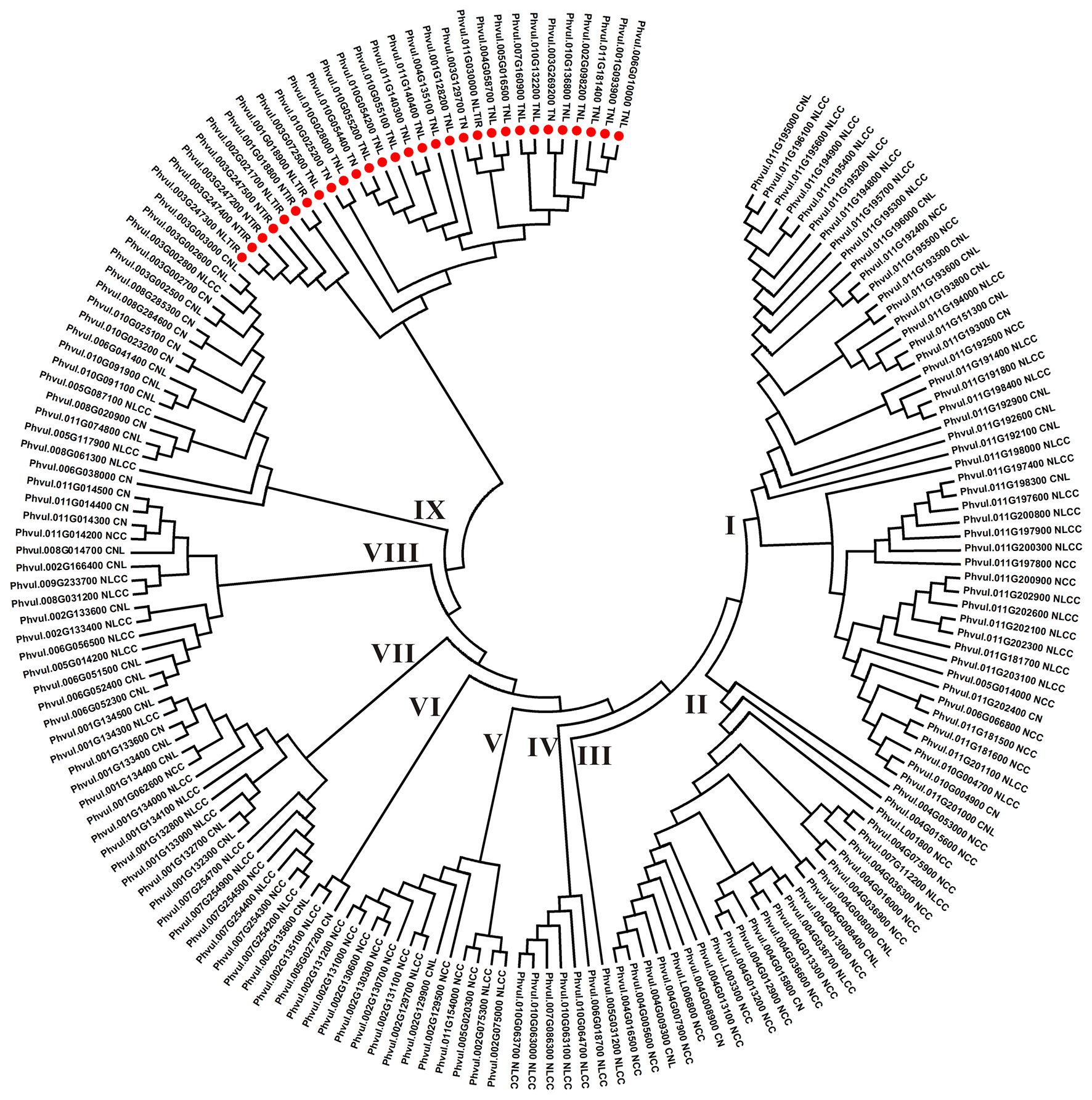

Supplement: Figure S2 — Heat map of the expression profiles of the NBS-LRR genes across different tissues. The expression data were downloaded from the Phytozome web site. The color scale shown below the heat map represents the expression values (FPKM), with green indicating low levels, and red indicating high levels of transcript abundance. [file Image2.TIF]

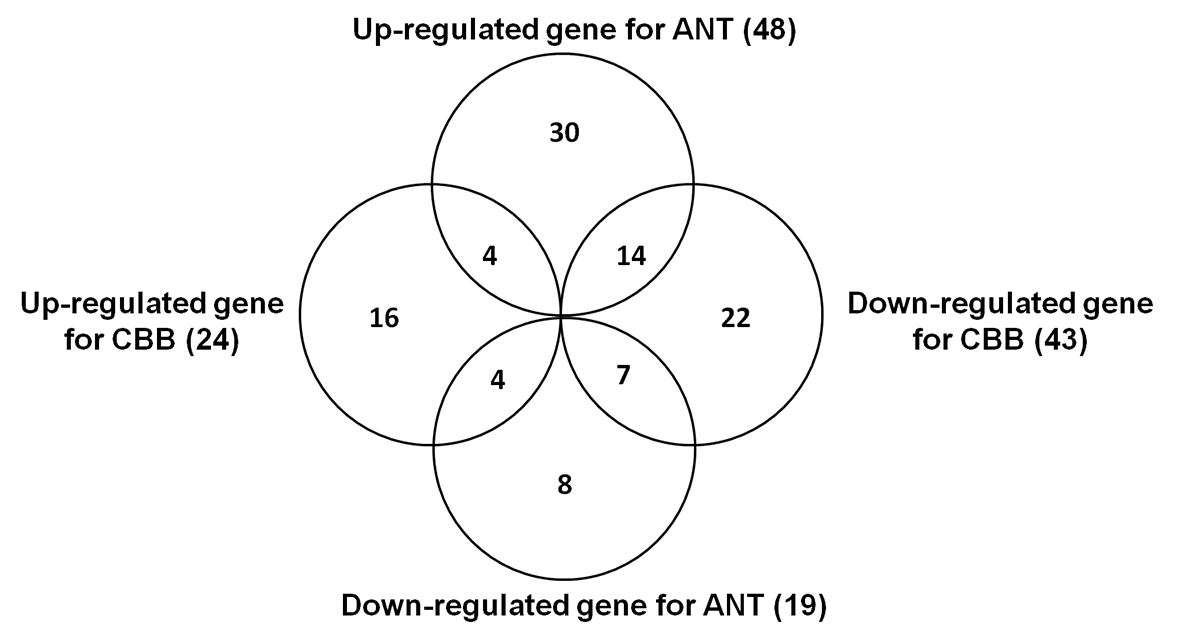

Supplement: Figure S3 — Number of differentially expressed genes for ANT and CBB. The numbers of differentially expressed genes that were exclusively up- or down-regulated for one disease are shown in each circle. The numbers of genes with common or opposite tendency of expression changes between different diseases are shown in the overlapping regions. The total numbers of up- or down-regulated genes for each disease are shown outside of the circles. [file Image3.TIF]

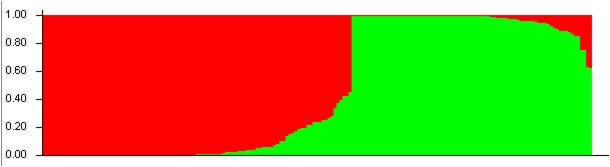

Supplement: Figure S4 — Estimated population structure of 183 common bean accessions (K = 2). Each group (subpopulation) is represented by a different color. [file Image4.JPEG]
